# Short-fiber Reinforced MOD Restorations of Molars with Severely Undermined Cusps

**DOI:** 10.3290/j.jad.b4051477

**Published:** 2023-04-25

**Authors:** Pascal Magne, Taban Milani

**Affiliations:** a Director, Center for Education and Research in Biomimetic Restorative Dentistry, Beverly Hills, CA, USA. Idea, hypothesis, experimental design, wrote the manuscript, proofread, submitted.; b Dental Student, Herman Ostrow School of Dentistry, University of Southern California, Los Angeles, CA, USA. Experimental design, performed the experiments, wrote in part the manuscript, proofread, performed certain tests, contributed substantially to discussion.

**Keywords:** short-fiber reinforced composite, composite resin, CAD/CAM, fatigue resistance, crack propensity, undermined cusps, shrinkage stress.

## Abstract

**Purpose:**

To assess the mechanical performance and enamel-crack propensity of large MOD composite-resin restorations on maxillary molars with severely undermined cusps.

**Materials and Methods:**

Thirty-six extracted maxillary third molars (n = 12) received a standardized slot-type MOD preparation (5-mm depth by 5-mm bucco-palatal width) with severe undercuts, leaving unsupported buccal and lingual enamel cusps. A short-fiber reinforced composite resin base (SFRC, everX Flow, GC) was used for both the experimental direct approach and semi-direct CAD/CAM inlays (Cerasmart 270, GC). In the control group using a direct approach, Gradia Direct (GC) composite resin was used alone without SFRC. Optibond FL (Kerr) adhesive was used in all three groups (also for the immediate dentin sealing of inlays). Artificial masticatory forces were simulated under water using closed-loop servo-hydraulics (MTS Acumen 3). Each specimen was mounted at a 30-degree angle and positioned so that a cylindrical antagonistic cusp (actuator) contacted the internal palatal cusp slope of the restoration. Cyclic loading was applied at a frequency of 5 Hz, starting with a load of 200 N, increasing by 100 N every 2000 cycles. Samples were loaded until fracture and the number of endured cycles and failure modes of each specimen was recorded. Each sample was also evaluated for crack propensity during the experiment and for final failure mode (reparable failures above the CEJ [cementoenamel junction] vs irreparable failures below the CEJ).

**Results:**

Shrinkage-induced cracks (>3 mm) were found in most specimens for both direct groups (66% to 83%) but not with inlays. The survival of inlays with a SFRC base was superior to that of the direct SFRC restorations and Gradia Direct (control) restorations (Kaplan-Meier survival analysis and post-hoc log-rank test p < 0.000). The direct control group without SFRC exhibited not only the poorest survival but also 100% catastrophic failure (vs 42% and 17% for SFRC direct and SFRC inlays, respectively).

**Conclusion:**

Large MOD restorations with severely undermined cusps were most favorably restored with an SFRC base and a CAD/CAM inlay, yielding the highest survival rate, more reparable failures and absence of shrinkage-induced cracks. When a low-cost restoration must be chosen, the SFRC base will significantly improve the performance and failure mode of directly layered restorations.

Large direct restorations in the posterior dentition present numerous challenges, including optimizing shape, contours and occlusal anatomy/function,^24,28^ in addition to the problem of polymerization shrinkage and contraction stresses.^6,26^ When strong adhesives are used, shrinkage may cause cuspal deformation and cracking of the enamel at the cusp base.^2,21^ When choosing direct techniques, it is paramount to reduce the contraction stresses.^5^ A number of techniques have been proposed to limit the effects of resin shrinkage, such as sophisticated layering techniques,^8^ sandwich approaches with glass-ionomer bases^22^ and fiber patches,^2^ pulse delay, and slow-start light polymerization protocols.^33^

An inescapable fact is that the majority of the shrinkage stress is developed during and after the vitrification stage, even in the absence of light (“dark-cure” stage). This does not permit stress relaxation on the time scales proposed for “soft” polymerization protocols,^19^ especially if a clinically relevant conversion rate is expected. Sandwich restorations using glass-ionomer bases constitute a very convenient means of reducing part of the shrinkage stresses (reducing the volume of shrinking material, or V-factor), especially when used in form of the novel “super-closed” technique.^8^ However, layering does not necessarily decrease shrinkage stresses^13,27^ and might even make them worse compared to bulk filling,^32^ probably because the V-factor is unchanged.

In the last decade, manufacturers have focused on the simplification of direct techniques (bulk filling). A new material (everX Posterior, GC; Tokyo, Japan) made of short-fiber reinforced composite (SFRC) was introduced in 2013 and has been recommended for high-stress bearing areas.^11^ It presents high flexural modulus and fracture toughness within the family of bulk-fill materials (12.6 GPa and 2.6 MPa m^1/2^, respectively), which far exceeds the properties of previous SFRCs. everX Posterior can be used easily in 4-mm-thick increments and can potentially match the toughness of dentin.^1,4^ When used as a dentin replacement in large posterior MOD restorations, it was able to improve the performance of direct restorations to that of CAD/CAM inlays.^30^ The difficult handling (high viscosity due to the 5%-15% content by weight of 0.017 x 0.8 mm e-glass fibers) and limited esthetics (high translucency) of everX posterior prompted the manufacturer to produce a flowable and more esthetic version (dentin opacity available) in 2019 called everX Flow (GC).^17^ Because of its smaller e-glass fibers (0.006 x 0.14 mm), this most recent version presents a higher fiber content (25% by weight) and superior fracture toughness (2.8 MPa m^1/2^) but lower flexural modulus (9.0 GPa) than everX Posterior. A remaining concern about everX Flow is the increased shrinkage stress compared to other SFRCs.^18^

Absolute control of polymerization stresses in large MOD restorations, however, is only possible through the use of inlay techniques,^7,8^ simply because of their extremely low V-factor (shrinkage is limited to the very thin layer of luting material). Composite resin CAD/CAM materials have gained popularity during the last decade because they demonstrate outstanding performance, wear properties, color integration, and millability in thin layers.^9,15,20,29,31^

This study assessed the accelerated fatigue strength and enamel-crack propensity of MOD direct composite restorations of molars with severely undermined cusps (with and without SFRC) compared to CAD/CAM composite resin inlays with SFRC. The null hypotheses were that (1) no significant difference in mechanical performance would be found between the restorative techniques used, and (2) there would be no difference in enamel-crack propensity (induced by shrinkage stress) between the three groups.

## Materials and Methods

Upon approval by the Ethics Review Committee of the University of Southern California (Los Angeles, CA) (proposal #HS-21-00568), 36 caries-free maxillary third molars without signs of occlusal wear were collected from an oral surgery clinic, scaled, pumiced, and stored in 0.1% thymol solution (Aqua Solutions; Deer Park, TX, USA). Only specimens with few or no cracks were chosen.

The roots were embedded up to 3 mm below the cementoenamel junction (CEJ) using acrylic resin (Palapress vario, Heraeus Kulzer; Hanau, Germany) and mounted in a stainless-steel positioning jig. The process of “enamel-crack tracking” was carried out during the whole experiment, in which each surface of the tooth was photographed under standardized conditions at 1.5X magnification (Nikon Z50 with a Nikkor 85-mm macro lens) using transillumination (IL-88-FOI Microscope Light Source, Scienscope; Chino, CA, USA). A new set of images was taken after 24 h and at 7 days post-restoration to detect new cracks.

In order to evenly distribute the teeth according to their size and shape, all specimens were organized into groups of three (triplets with similar buccolingual and mesiodistal dimensions) and subsequently randomly re-assigned to groups (n = 12) which received 1. a layered direct composite (Gradia Direct, GC); 2. a fiber-reinforced composite resin base (dentin-shade everX Flow, GC) layered with direct composite (Gradia Direct); 3. a fiber-reinforced composite resin base (dentin-shade everX Flow) and a CAD/CAM inlay (Cerasmart 270, GC).

### Tooth Preparation

**Fig 1:**
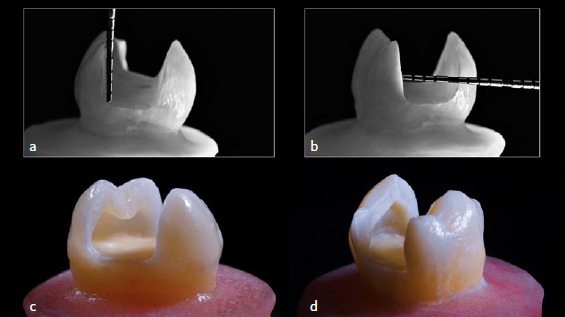
Standard MOD tooth preparation and corresponding measurements: (a) 5 mm deep; (b) 5 mm in bucco-palatal width, and severely undermined cusps with unsupported enamel; (c) lingual view; (d) buccal view.

A high-speed electric handpiece and tapered diamond burs (Brasseler; Lemgo, Germany) were used to prepare a standardized MOD slot-type defect with 5-mm bucco-palatal width and 5-mm depth. A round diamond bur (801-014 and 801-010) was used to remove all the dentin from underneath buccal and lingual cusps, creating a severe undercut, undermining the enamel to a residual thickness of 1 mm (measured with a Precision Metal Caliper, Buffalo Dental; Syosset, NY, USA). A 0.5- to 1-mm 45-degree bevel at the cervical and proximal angles was created with a spherical fine-diamond bur for direct restorations only. After preparation completion (Fig 1), photographic enamel-crack tracking was performed to determine if preparation caused any damage to the specimens.

For the CAD/CAM inlay preparations, immediate dentin sealing (IDS) was performed on the freshly cut dentin using a three-step etch-and-rinse dentin adhesive (Optibond FL, Kerr; Orange, CA, USA) according to a standardized protocol.^23^ Etching and bonding during IDS was extended to the internal undermined enamel. The adhesive was polymerized for 20 s at 1000 mW/cm^2^ (VALO Curing Light, Ultradent; South Jordan, UT, USA) followed by the placement of dentin-shade everX Flow to fill the undercuts and create a 1-mm coverage of the pupal floor. This was then polymerized for 20 s and an additional 10 s under an air-blocking barrier (KY Jelly, Johnson & Johnson; Montreal, QC, Canada). The enamel margins were re-finished with a spherical fine-diamond bur (Brasseler) to remove excess adhesive resin.

### Restorative Procedures

All inlays were fabricated with the Cerec CAD/CAM system (Dentsply Sirona; Konstanz, Germany) and restorations were designed using the Cerec 4.4 software. To improve standardization, the original design of the restoration was not edited; only the position tools were used to ensure correct thickness. Composite resin blocks (Cerasmart 270, GC) were milled, carefully adjusted under a microscope (Leica MZ 125, Leica Microsystems; Wetzlar, Germany), and mechanically polished. The fitting surface of all restorations was air abraded (RONDOflex plus 360, KaVo; Biberach, Germany) using 30-µm silica-modified aluminum oxide (Rocatec Soft, 3M Oral Care; St Paul, MN, USA) for 10 s at a distance of 10 mm and a pressure of 30 psi, followed by immersion in distilled water in an ultrasonic bath for 2.5 min and air drying. Silane (Ultradent) was applied for 20 s, air- and heat-dried at 100°C for 1 min (D.I.-500, Coltene; Altstätten, Switzerland). The prepared tooth surface was air abraded to clean and reactivate the IDS layer using 30-µm silica-modified aluminum oxide, followed by etching for 30 s with 35% phosphoric acid (Ultra-Etch, Ultradent) and abundant rising and drying. Adhesive resin (Optibond FL Adhesive, Kerr) was applied to both surfaces (tooth and inlay) and left unpolymerized until the luting material (Gradia Direct, GC) – preheated for 5 min in a Calset warmer (AdDent; Danbury, CT, USA) – was inserted into the preparation, followed by complete seating of the inlay. Composite resin excess was removed, and each surface was light polymerized for a total of 60 s (20 s per surface, repeated 3 times), with an additional 10 s under an air-blocking barrier. The margins were mechanically polished.

**Fig 2:**
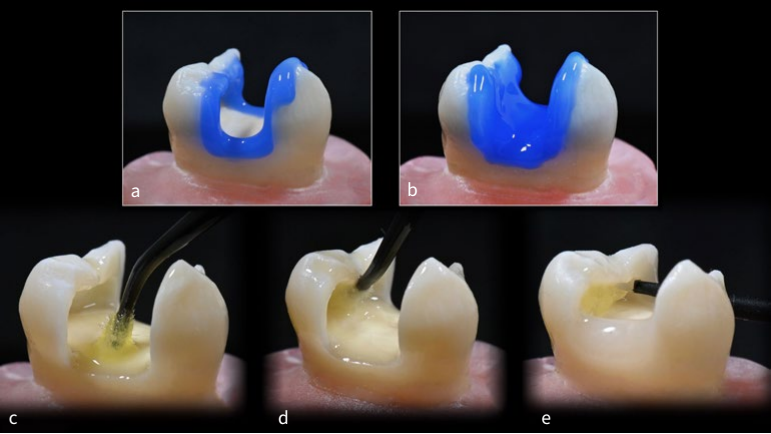
Direct SFRC steps: (a) enamel etching with 37.5% phosphoric acid, extended 1 mm beyond the bevel into the enamel for 20 s before extending into (b) dentin for an additional 10 s (rinsed abundantly and gently air dried). (c) Application of 2-3 coats of Optibond FL Primer to the etched dentin surface with light scrubbing motion for at least 15 s, followed by gentle air drying; (d) coating with Optibond FL Adhesive applied to etched enamel and primed dentin surfaces with light scrubbing motion for 15 s (excess removed from margins using a dry microbrush), followed by light polymerization for 20 s and (e) SFRC placement for approximate dentin replacement (including undercuts) with flowable composite resin (EverX Flow) and light polymerization for 20 s. Note that the proximal boxes were elevated first with one composite increment (Gradia Direct), in order to prevent SFRC from slumping near the gingival margins.

**Fig 3:**
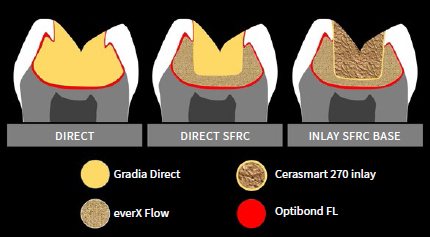
Schematic representation of restorative design for each experimental group.

For SFRC direct composite restorations (Fig 2), dentin and enamel were bonded using the same three-step etch-and-rinse adhesive (Optibond FL; Kerr), which was light polymerized for 20 s at 1000 mW/cm^2^ (VALO Curing Light). A standardized natural layering technique was applied in seven increments. The proximal walls were raised with two 2-mm-thick enamel-shade increments (Gradia Direct, GC). Approximately 2.5-mm of the remaining Class-I defect (including the undercuts) was filled using fiber-reinforced composite (dentin-shade everX Flow) and light polymerized. Final layering was performed using 3 increments that were individually polymerized, first the floor, followed by the enamel cusps (Fig 3d). Special attention was given to strictly emulating the cuspal inclination and occlusal anatomy of the CAD/CAM inlays previously designed. Each increment was polymerized for 20 s at 1000 mW/cm^2^, and final light polymerization was performed for 10 s under an air-blocking barrier (KY Jelly, Johnson & Johnson). Finishing procedures were the same as for the previous group.

The same technique was used for the direct control group, except SFRC was substituted by 3 increments of conventional composite resin (Gradia Direct, GC), for a total of 10 increments for the whole restoration. The restorative design for each group is presented schematically in Fig 3.

### Accelerated Fatigue Test

**Fig 4:**
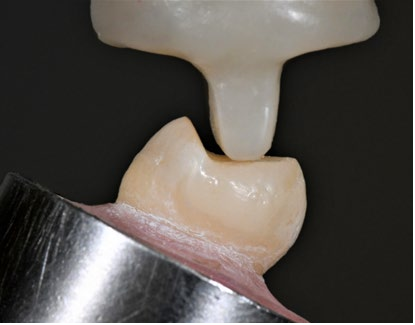
Positioning of specimen for accelerated fatigue test at a 30-degree angle. The semi-cylindrical antagonist cusp is made of microhybrid-spheroidal composite resin and contacts the center of the palatal cusp slope.

Restored specimens were kept in distilled water at ambient temperature for 1 week. Enamel crack tracking by transillumination and photography was performed for each tooth surface and followed by the fatigue test in an artificial mouth using a closed-loop electrodynamic system (Acumen 3, MTS Systems; Eden Prairie, MN, USA). All fatigue tests were continuously recorded and monitored using transillumination and a macro video camera (Canon Vixia HF S100, Canon USA; Melville, NY, USA). The masticatory forces were simulated through a composite-resin cusp (Filtek Z100, 3M Oral Care) shaped in a semicylinder (2.5-mm radius) contacting the center of the palatal cusp slope. The cusp slope was prepared flat, and the load point was equidistant from the palatal cusp tip and the central groove. Isometric contraction forces (load control) were applied at a 30-degree angle to the tooth’s long axis (Fig 4). The load chamber was filled with distilled water to submerge the sample during testing. A cyclic load was applied at a frequency of 5 Hz, starting with a load of 200 N and increasing by 100 N every 2000 cycles. Samples were loaded until fracture and the number of endured cycles and failure modes of each specimen was recorded. Specimens were loaded until fracture and the number of endured cycles was registered. After the test, each sample was evaluated by transillumination and optical microscopy (Leica MZ 125; Leica Mycrosystems) at a 10:1 magnification (two-examiner agreement).

### Enamel-Crack Tracking

To detect new enamel cracks, specimens were evaluated 3 times during the experiment at 1.5X magnification (Nikon Z50 with a Nikkor 85-mm macro lens) under standardized conditions with transillumination (IL-88-FOI Microscope Light Source, Scienscope): before and 24 h after tooth restoration and 1 week after restoration. In cases of doubt, a two-examiner agreement was sought and analyzed under an optical microscope at 10:1 magnification (Leica MZ 125, Leica Microsystems). Special care was taken to differentiate between pre-existing cracks from those created by polymerization shrinkage. Cracks were classified into 3 categories based on previous studies:^2,22^ (a) no cracks visible, (b) visible cracks smaller than 3 mm, and (c) visible cracks larger than 3 mm.

### Statistical Analysis

The fatigue resistance of the groups was evaluated using the Kaplan-Meier analysis (survived cycles) for the accelerated fatigue test. The post-hoc log-rank test was used to compare the influence of the restorative procedure on the fatigue resistance of the teeth at a significance level of 0.05. The data were analyzed with statistical software (SPSS 23, SPSS; Chicago, IL, USA).

## Results

**Fig 5:**
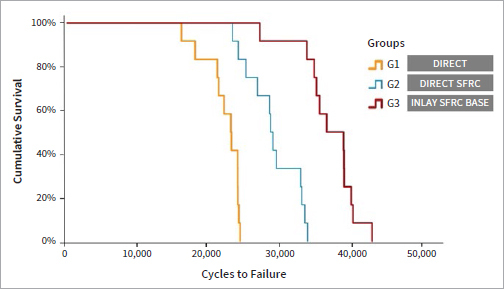
Kaplan-Meier survival curves.

Survival after the accelerated fatigue test was significantly different for all 3 groups (Fig 5, Table 1, Kaplan-Meier followed by the log-rank test, p>0.000). The best performance was documented for inlays with an SFRC base, followed by direct SFRC composite resin restorations. The direct control group had the worst survival rate.

**Table 1:** Fatigue-to-failure pairwise post-hoc comparisons

	Direct control	Direct SFRC	Inlay SFRC base
Direct control	–	0.000*	0.000*
Direct SFRC	0.000*	–	0.000*
Inlay SFRC base	0.000*	0.000*	–

Gray background: resistance to fatigue post-hoc tests for cycles (Kaplan-Meier followed by log-rank test). Colorless background: resistance to fatigue post-hoc tests for load (Life Table followed by Wilcoxon-Gehan test). *Statistically significant difference between groups (p<0.05).

**Fig 6:**
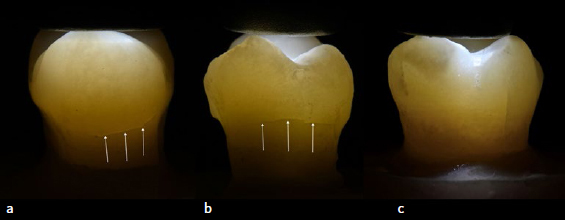
Example of visible cracks >3 mm using transillumination tracking in all three groups. (a) Gradia Direct posterior composite resin; (b) SFRC base with Gradia Direct posterior both exhibiting large shrinkage-induced cracks; (c) CAD/CAM inlay with SFRC base (vertical crack existed prior to restoration).

No new cracks were observed after tooth preparation. Figure 6 and Table 2 show that after restoration and 1 week of water storage, the crack propensity was higher for the direct control group (83%), followed by the direct SFRC (66%) and CAD/CAM inlay groups (0% of shrinkage-induced cracks larger/smaller than 3 mm).

**Table 2:** Crack propensity after 1 week of restorative procedures and before accelerated fatigue test

Group	No cracks	Cracks <3 mm	Cracks >3 mm
Direct control (n = 12)	0	2 (17%)	10 (83%)
Direct SFRC (n = 12)	0	4 (33%)	8 (67%)
Inlay SFRC base (n = 12)	11 (92%)	1 (8%)	0

**Fig 7:**
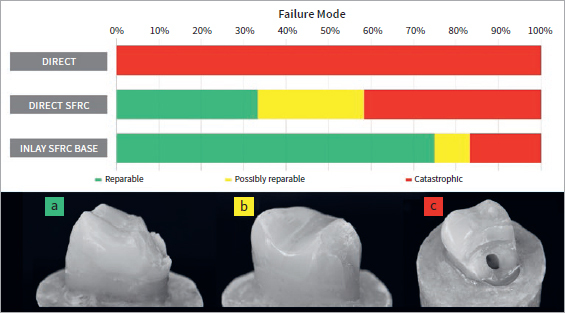
Failure mode distribution for each group (top) and an exemplary specimen for each failure mode: (a) reparable; (b) possibly reparable; (c) catastrophic.

Failure mode was evaluated with the aid of transillumination and magnification to classify the fracture as reparable, possibly reparable (below the CEJ but above the acrylic resin base, possibly reparable with additional interventions such as margin elevation or periodontal surgery), and irreparable. Irreparable failure, i.e. below the acrylic resin-base limit, affected 100% of the direct control specimens, but ranged between 17% and 42% for the inlay with SFRC base and direct SFRC composite resin groups, respectively (Fig 7, Table 3).

**Table 3:** Failure types, numbers, and percentages after fatigue-to-failure test

Group	Reparable (above CEJ)	Possibly reparable (below CEJ above ARB)	Irreparable (below ARB)
Direct control (n = 12)	0	0	12 (100%)
Direct SFRC (n = 12)	4 (33%)	3 (25%)	5 (42%)
Inlay SFRC base (n = 12)	9 (75%)	1 (8%)	2 (17%)

CEJ: cementoenamel junction; ARB: acrylic resin base.

## Discussion

The present study assessed the accelerated fatigue strength and enamel-crack propensity of MOD direct composite restorations of molars with severely undermined cusps (with and without short-fiber reinforced composite) compared to CAD/CAM composite resin inlays with SFRC. The null hypotheses are rejected because (1) a significant difference in mechanical performance and failure mode between the restorative techniques was found, and (2) enamel-crack propensity (induced by shrinkage stress) was not the same across all groups.

This in-vitro study allowed a high level of standardization for all procedures by controlling tooth dimensions, exact preparation dimensions, loading steps, loading configuration, and occlusal morphology. The innovative randomly reassigned multiplets method described in the material and methods section was part of this effort to eliminate confounding variables. Such level of standardization would simply be impossible in a clinical study, in which the number of confounding variables is such (patients’ masticatory and dietary habits, individual caries susceptibility, as well as the need for multiple operators and evaluators, etc.) that differences between groups are often masked. True fatigue tests at low loads and high cycles are extremely time-consuming because more than 1,000,000 cycles are necessary before observing any failure.^14^ The accelerated fatigue test used in this work, originally introduced by Fennis et al,^10^ is therefore the most relevant means of assessment because it replicates the clinical mode of failure in a reasonable amount of time. The Acumen 3 (MTS) electrodynamic system used here features a rigid load frame and a direct-drive linear motor providing highly precise load and motion control. Previously published methods and load protocols from works comparing large direct and CAD/CAM MOD restorations performed at the same facility^2,22,30^ were used. The angle of force was modified to 30 degrees and applied to the supporting cusp using a composite resin cylinder (Filtek Z100; 3M Oral Care) as an antagonist. This increased the stress to the restoration and simulated an extreme load scenario (non-working contact).

Extreme loads were used (far in excess of physiological masticatory forces), and all specimens survived the first half of the experiment (up to 1000 N), demonstrating outstanding survival rates. Despite the severe undermining of the cusps and unsupported enamel, the results of the present study align with previously published works performed at the same facility, in which large MOD Filtek MZ100 CAD/CAM inlays,^22^ Cerasmart CAD/CAM inlays, and Gradia Direct semi-direct inlays^30^ yielded the best performance. Cerasmart 270 is filled with 78wt% silica- and barium-glass nanoparticles, while MZ100 is filled with 85wt% spheroidal zirconia-silica nanofillers. The same adhesive (Optibond FL; Kerr) and protocol was used in all those experiments, including the immediate dentin sealing technique (IDS),^23^ which may also account for the high performance achieved. In fact, in a study by Hofsteenge et al,^12^ inlays with IDS (Optibond FL) and overlays without IDS did not differ in terms of fracture strength, in addition to which inlays had always more favorable fracture modes than onlays.

The direct techniques in the present experiment were not able to match the performance of the CAD/CAM inlay (for both accelerated fatigue and crack propensity), as was the case for one of the comparable experiments (including sandwich techniques and the use of fiber patches). The use of the everX Flow short-fiber reinforced dentin base, however, was able to improve the fatigue resistance and failure mode within the direct groups. The extreme loads required to fracture the restored teeth speak for the ability of the fiber-reinforced base to function in a high-stress–bearing area^11^ and its potential ability to match the toughness of dentin.^1,4^ However, the flowable SFRC base (everX Flow) had only a limited effect on crack propensity. This effect was very significant when using the original SFRC (everX Posterior) in a comparable experiment,^30^ in which no shrinkage-induced cracks >3 mm were found even using the direct technique (without undermining the cusps). This is explained in part by the significant difference in polymerization volumetric shrinkage and shrinkage stress between the original everX Posterior (-1.52%) and the newer, flowable version everX Flow (-2.58%).^17,25^ Absence of shrinkage-induced cracking is not a surprise with inlays; however, in direct techniques, the very limited incidence of cracks reflects the good performance of everX Posterior. The flowable composite everX Flow was developed with the idea of facilitated placement, which was obtained at the price of the fiber length:width critical-aspect ratio (20-30 instead of 70 for the original everX Posterior). This allowed increasing the fiber content to 25% (instead of 5%-15% for the original), but also required a modification of the resin matrix and reduction in barium-glass filler content, possibly explaining the increased shrinkage rate.

It is the essence of BRD (Biomimetic Restorative Dentistry) to mimic tooth structure and as such, SFRCs constitute the most biomimetic dentin replacements because of their superior fracture toughness. Natural dentin is reinforced by collagen fibers that can stop and deflect cracks initiating in enamel. The future of SFRCs might lie in the combination of millimeter- and micrometer-scale fibers with aspect ratios of 20-70, the so-called “hybrid SFRC”. Preliminary results by Lassila et al^16^ are extremely encouraging: experimental “hybrid” composite resins reinforced with different fiber lengths had statistically significantly greater mechanical performance in terms of fracture toughness (4.7 MPa m^1/2^) and flexural strength (155 MPa) compared to other tested composites.

In agreement with existing data obtained under the same conditions,^22,30^ it can be stated that the SFRC direct and CAD/CAM inlay restorations also presented more favorable failure types. No catastrophic failures were observed in Cerasmart 270 inlays with a SFRC base, as was shown in the previous studies when using MZ100 inlays.^22,30^ Among the limitations of the present study was the absence of a Cerasmart 270 inlay group without SFRC (inlay control group). The SFRC base can primarily be considered a crack-arresting layer or dentin replacement. Thus, its thickness might contribute to the fatigue performance and failure mode. In the present study, the SFRC was applied in a relatively thin layer of 1-2.5 mm instead of a bulk or core layer. Further studies should consider thicker layers or even a full restoration made of SFRC, the limitation of which could be the surface polishing and surface degradation due to the exposition of the fibers.

There are two important outcomes of this study. The first is the combination of absence of major cracks induced by shrinkage, best fatigue performance, and most favorable failure types for the inlays with SFRC base. While cost effectiveness for the patient might be a limiting factor, from a clinical standpoint, it is undeniable that occlusion and morphology are optimized with inlays rather than direct techniques. Second, the positive effect of the SFRC base on the shrinkage, performance, and failure mode of direct restorations must be mentioned. Directly layered restorations constitute a viable alternative due to the simplicity of the procedure, not just because it is an inexpensive technique.

## Conclusions

The challenge of restoring large MOD defects with severely undermined cusps was assessed using 3 restorative approaches (direct with and without SFRC base, and CAD/CAM inlay with a SFRC base). Restorations with SFRC bases yielded excellent mechanical performance even above physiological masticatory loads. Large MOD defects, however, are best restored using CAD/CAM inlays with an SFRC base for optimal strength, reduced shrinkage-induced cracks, and most favorable failure mode. When a low-cost restoration must be chosen instead, the SFRC base will significantly improve the performance and failure mode of directly layered restorations.

## Clinical relevance

The use of an SFRC base is highly recommended when restoring molars with severely undermined cusps and large MOD defects, whether a direct (low-cost) or semi-direct CAD/CAM inlay is chosen. However, CAD/CAM inlays seem to perform better than direct techniques.
